# Effective Generation of Functional Pancreatic β Cells from Human-Derived Dental Stem Cells of Apical Papilla and Bone-Marrow-Derived Stem Cells: A Comparative Study

**DOI:** 10.3390/ph16050649

**Published:** 2023-04-26

**Authors:** Duaa Abuarqoub, Sofia Adwan, Rand Zaza, Suha Wehaibi, Nazneen Aslam, Hanan Jafar, Nidal Qinnah, Abdalla Awidi

**Affiliations:** 1 Department of Pharmacology and Biomedical Sciences, University of Petra, Amman 11196, Jordan; 2 Cell Therapy Center, The University of Jordan, Amman 11942, Jordannazneenaslam88@hotmail.com (N.A.);; 3 Department of Medical Laboratories, Faculty of Health Sciences, American University of Madaba, Madaba 11821, Jordan; 4 School of Medicine, The University of Jordan, Amman 11942, Jordan; 5 University of Petra Pharmaceutical Center (UPP), University of Petra, Amman 11196, Jordan; 6 Department of Internal Medicine, Jordan University Hospital, Amman 11942, Jordan

**Keywords:** diabetes, stem cells, dental stem cells, bone marrow, β cells, differentiation

## Abstract

Diabetes Mellitus Type 1 is an autoimmune disease that occurs due to the destruction of insulin-producing cells (β cells), resulting in hyperglycemia. Therefore, diabetic patients depend on insulin treatment for the rest of their lives. Stem cells are considered a promising cellular therapy to replace the nonfunctional beta cells with functional and mature beta cells. Hence, in this study, we aimed to examine the potential of dental stem cells of apical papilla (SCAP) to differentiate into functional islet cell aggregates (ICAs), compared to the ICA generated from bone-marrow-derived stem cells (BM-MSCs). Our strategy was to induce the differentiation of SCAP and BM-MSCs into a definitive endoderm. The success of endodermal differentiation was determined by measuring the expression of definitive endodermal markers, FOXA2 and SOX-17, by flow cytometry. Next, the maturity and functionality of the differentiated cells were evaluated by measuring the amount of insulin and C-peptide secreted by the derived ICAs using ELISA. Additionally, the expression of mature beta cell markers—insulin, C-peptide, glucagon and PDX-1—was detected through confocal microscopy, while the staining of the mature islet-like clusters was detected by using diphenythiocarbazone (DTZ). Our results have shown that both SCAP and BM-MSCs were sequentially committed to a definitive pancreatic endoderm and β-cell-like cells by upregulating the expression of FOXA2 and SOX17 significantly (**** *p* < 0.0000 and *** *p* = 0.0001), respectively. Moreover, the identity of ICAs was confirmed by DTZ-positive staining, as well as by the expression of C-peptide, Pdx-1, insulin and glucagon at day 14. It was noted that at day 14, differentiated ICAs released insulin and C-peptides in a significant manner (* *p* < 0.01, *** *p* = 0.0001), respectively, exhibiting in vitro functionality. Our results demonstrated for the first time that SCAP could be differentiated into pancreatic cell lineage in a similar manner to BM-MSCs, suggesting a new unambiguous and nonconventional source of stem cells that could be used for stem cell therapy to treat diabetes.

## 1. Introduction

The unique β cells in the pancreatic islets of Langerhans are required for the production of all the insulin needed for the glucose homeostasis of any organism [1]. Plasma insulin levels are principally determined by the mass of insulin-producing beta cells (IPCs) in these islets and the output of each of these beta cells—namely, the beta cell mass and function, respectively. Hence, defects in either beta cell mass or function, or both, can result in inadequate levels of plasma insulin that can effectively lower plasma glucose concentrations, leading to the onset of hyperglycemia and diabetes [2]. Diabetes Mellitus (DM) can be caused by an absolute insulin deficiency due to the autoimmune destruction of IPCs (T1DM) or by insulin resistance and relative insulin deficiency [3]. IPC transplantation was once considered as a promising therapeutic approach for T1DM by restoring normoglycemia in diabetic patients [4]. Nonetheless, one of the drawbacks of this strategy is the limited resources of human islet tissues, the shortage in number of donors and their immunological matching [5]. Consequently, investigating and searching for stem cells that have the potential to differentiate into IPCs, boosting pancreatic regeneration, and improving insulin sensitivity are all considered as alternatives to the islet cell transplant approach [6,7].

Mesenchymal stem cells (MSCs) possess a differentiation capacity, anti-inflammatory effects, and immunosuppressive properties, thus they are considered as a gold-standard-candidate cell type for the treatment of DM [6]. Among the lineages that MSCs can differentiate to, MSCs isolated from different sources have shown success of differentiation into beta cells [8,9,10,11,12]. Moreover, stem-cell-based therapeutic approaches are very promising in future for treating acute and chronic pancreatitis by reducing the inflammation, apoptosis and fibrosis of pancreas, in addition to the reduction in the level of pancreatic damage [13]. Different studies have shown the role of different types of MSCs in treating pancreatitis, resulting in a decline in pancreatic damage, regenerating amylase cells and retaining the morphological appearance of the pancreatic cells as well [14,15].

MSC differentiation into beta cells goes through two major steps. First, MSCs are differentiated into pancreatic progenitors using mainly nicotinamide augmented by L-Taurine and sodium butyrate followed by beta cell maturation. Their maturation to beta-like cells is obtained by nicotinamide blended with exendin-4 or glucagon-like peptide-1 (GLP-1) [16].

Thus, the correct reprogramming of MSCs to activate these pathways is crucial for inducing their differentiation into IPCs. The success of stem cells’ differentiation into IPCs depends on various factors such as the source of stem cells, supplements added into the differentiation medium, and the duration of the differentiation procedure [17].

The present protocols generate cells that exhibit some phenotypic resemblance of beta cells, such as expressing some of the critical beta cell markers. Nonetheless, further optimization is needed to induce mature, functional cells for therapy.

Although bone-marrow-derived MSCs (BM-MSCs) are the pioneer cells in the beta cell differentiation, the method of acquiring the sample is invasive. Therefore, more research is required to isolate and characterize MSCs from less invasive sources. One good example are stem cells from the apical papilla (SCAP), which are rich in source, easy to collect and non-invasively isolated [12]. Therefore, in this current study, we aim to compare the beta cell differentiation potential of SCAP producing insulin-producing cells (IPCs) to that of the bone-marrow-derived MSCs.

## 2. Results

### 2.1. Characterization of Human MSCs

**Figure 1:**
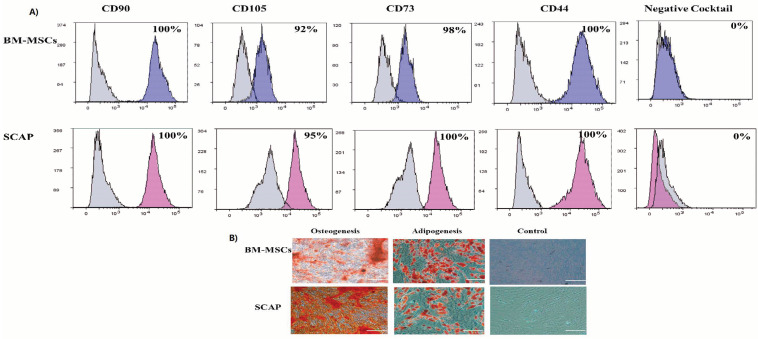
Characterization of human-derived stem cells BM-MSCs and SCAP. (**A**) Flow cytometric analysis of both SCAP and BMMSCs showing a positive expression of MSC surface markers: CD90, CD105, CD73 and CD44 and a negative expression of hematopoietic stem cell markers: CD34, CD45, CD11b, CD19 and HLA-DR. (**B**) Multilineage differentiation of both SCAP and BMMSCs into osteogenic differentiation and adipogenic differentiation, compared to the control uninduced cells (scale bar = 100 µm).

The derived BM-MSCs and SCAP cells showed typical mesenchymal stem cell markers as per the statement position of the International Society for Cellular Therapy (ISCT) [18]. Both cell types were positive for MSC surface markers; CD90, CD105, CD73 and CD44 and were negative for CD34, CD45, CD11b, CD19 and HLA-DR (Negative Cocktail) (Figure 1A). Additionally, when induced to differentiate, the derived cells were able to differentiate into adipocytes and osteocytes, thereby confirming their stemness characteristics (Figure 1B).

### 2.2. Validation of Definitive Endoderm Induction by Flow Cytometry

As a starting point, cells were checked to define their ability to form a definitive endoderm layer by adding definitive endoderm media to the derived cells.

**Figure 2:**
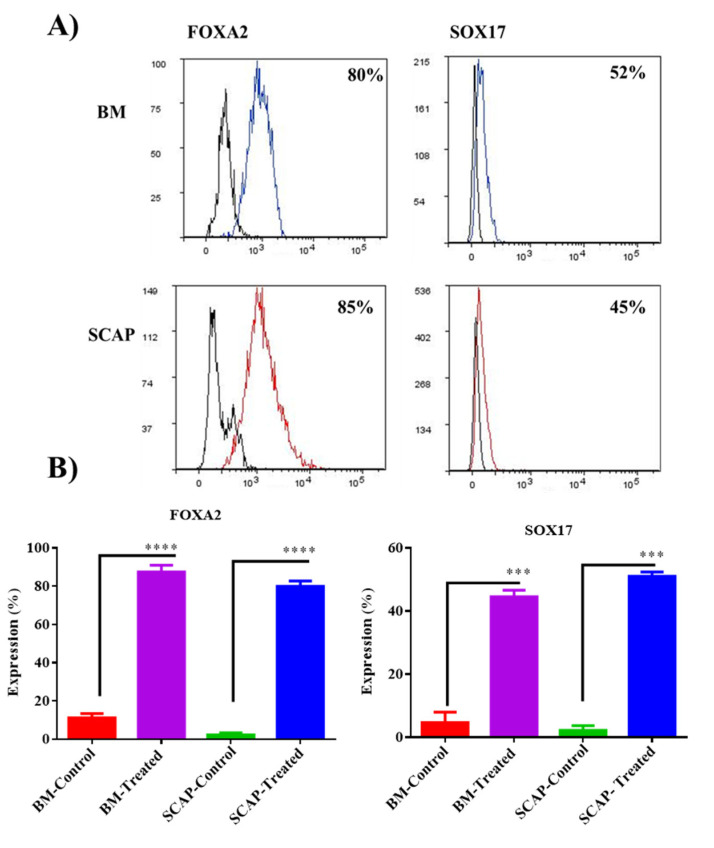
Definitive endoderm induction. (**A**) Flow cytometric analysis of the expression of definitive endoderm markers, SOX17 and FOXA2, after the induction of both BMSCs and SCAP by two different methods. (**B**) Statistical analysis of the expression of definitive endoderm markers, after the induction of human MSCs, SCAP and BMMSCs, into a definitive endoderm layer, SOX17 (*** *p* < 0.0001) and FOXA2 (**** *p* < 0.0001), compared to the control untreated groups.

The formation of the definitive endoderm was confirmed by measuring the expression of SOX17 and FOXA2 using flow cytometry. Induced cells showed an interesting expression of these two markers in comparison with the uninduced cells. For SOX17, both SCAP and BM adherent cells showed a significant increase in the expression of SOX17 (45% and 52%) compared to the control untreated cells, respectively (*** *p* < 0.0001). Additionally, FOXA2 was expressed in a high statistical manner in both induced groups SCAP (85%) and BM-MSCs (80%) compared to the control untreated group (**** *p* < 0.0001) (Figure 2). However, no significant difference was observed between BM-induced cells and SCAP-induced cells.

### 2.3. Diphenylthiocarbazone Staining (DTZ)

**Figure 3:**
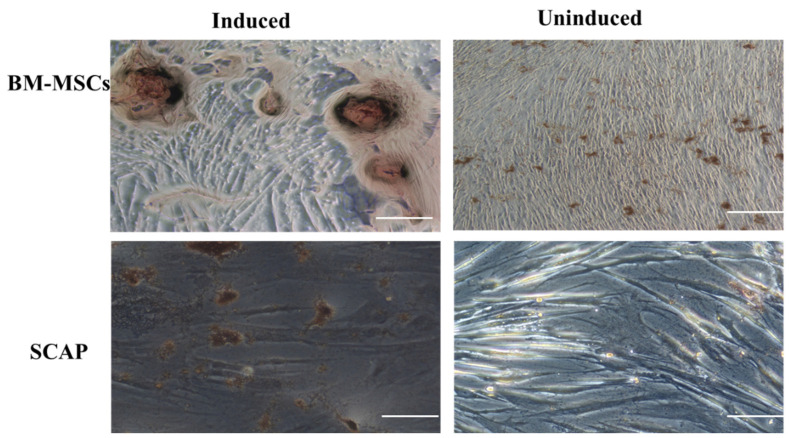
Diphenylthiocarbazone staining of islet-like clusters (ILCs) that are generated after 14 days of beta cell differentiation of SCAP and BM-MSCs as adherent cells resulted in the formation of islet-like clusters (400×).

At a later stage of differentiation, the formation of islet-like clusters was detected at day 14 of differentiation in the induced cells and the clusters were stained using DTZ dye. On the other hand, uninduced cells did not show any formation of the clusters (Figure 3).

### 2.4. Secretion of Insulin and C-Peptide by Differentiated Cells by ELISA

**Figure 4:**
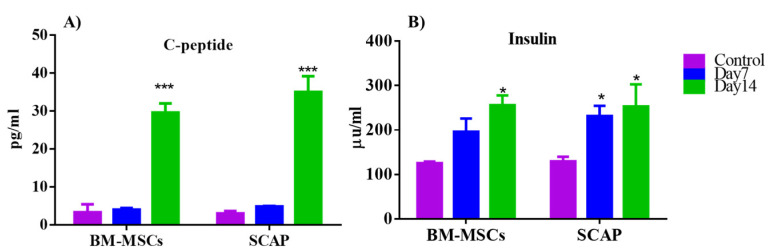
Measurements of the concentrations of the secreted markers, insulin and C-peptide, secreted by the differentiated cells, SCAP and BM, by two different methods by ELISA after 14 days of induction. (**A**) C-peptide, (**B**) insulin. *** *p* = 0.0001. * *p* < 0.05.

Both C-peptide and insulin were measured at days 1, 7 and 14 of differentiation. Interestingly, for C-peptide secretion, both SCAP and BM-MSCs showed a significant upregulation in the secretion level of insulin at day 14 of induction compared to that at day 1 (*** *p* = 0.0001). However, no significant difference was observed between day 1 and day 7 of C-peptide’s secretion (Figure 4). For insulin, after 14 days of induction, a significant elevation in the secretion level of insulin was recorded compared to day 1 for both SCAP and BM-MSCs (* *p* < 0.05). For SCAP-induced cells only, a significant increase was observed at day 7 of induction in comparison to day 1 (* *p* < 0.05).

On the other hand, no statistically significant difference in the secretion levels of BM and SCAP among differentiation days was recorded.

### 2.5. Validation of Islet-like Clusters Maturation Markers; Insulin, C-peptide, Glucagon and PDX 1 by ICC

**Figure 5:**
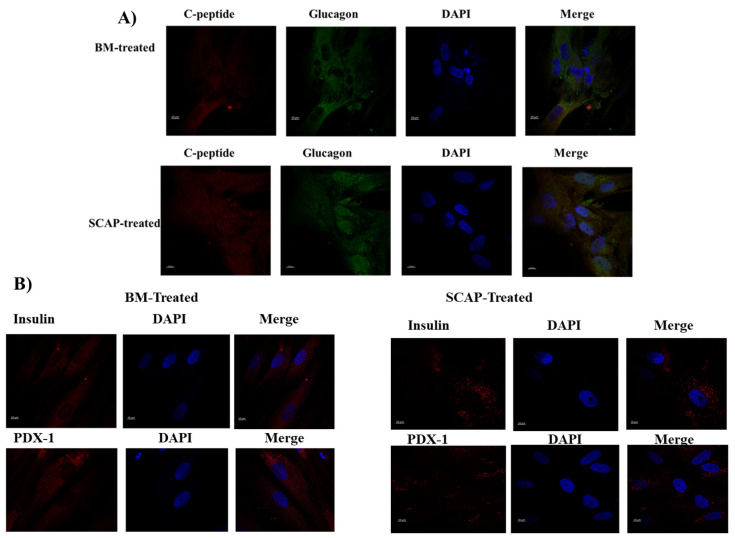
Immunofluorescence staining of pancreatic beta cell markers, (**A**) C-peptide and glucagon, (**B**) insulin and PDX-1, after 14 days of induction of SCAP- and BM-MSC-differentiated cells. Images were taken by a confocal microscope (25×).

The maturation of islet-like clusters was examined by studying the maturation markers (insulin, C-peptide, glucagon and PDX-1). These markers are highly expressed when islet cells become mature beta cells. Mature insulin-producing cells (IPCs) derived from BM-MSCs and SCAP were able to express these maturation markers—insulin, C-peptide, glucagon and PDX-1—after 14 days of differentiation in comparison with uninduced control cells (Figure 5).

## 3. Discussion

Isolation, expansion and differentiation of stem cells from a nonembryonic origin can provide an essential therapeutic cell source for regenerative medicine [19]. Many studies have demonstrated that different sources of MSCs can differentiate not only into mesenchymal but also can transdifferentiate into ectodermal and endodermal lineages [20]. MSCs can contribute to therapy for various clinical settings but they may exhibit variable functional properties depending on, but not restricted to, their source and cell culture conditions [21,22]. Of the studied MSC sources, BM-MSCs offer distinct advantages with respect to their availability and the extent of their documentation in published research [23].

In our present study, we compared stem cells differentiated from BM and apical papilla SCAP with regard to their β-differentiation potential and generation of mature insulin-producing cells.

In order to generate functional β cells from stem cells, there is a necessity to evaluate the stage of differentiation toward mature and functional β cells. In this study, cells isolated from bone marrow and apical papilla were initially characterized relative to their morphology, phenotypic characteristics and their multilineage differentiation ability. The expression of definitive endoderm markers (FOXA2 and SOX17) after the induction of the BM- and AP-derived cells into a definitive endoderm layer was also evaluated in this study. Definitive endoderm (DE) is described as a population of squamous cells that express FoxA2 and Sox17, formed after the formation of a primitive endoderm [24].

FoxA2 is the first gene to be activated from the FoxA family that are expressed at an early stage during the formation and establishment of definitive endoderm lineage [25]. Furthermore, SOX17 has been illustrated to specifically participate in the late stage of differentiation of extraembryonic endoderm towards visceral and parietal endoderm cells and is not involved in the early stage of differentiation [26]. In line with these findings, after 2 days of induction, we found that almost all induced cells from both sources showed a high expression pattern of FoxA2 marker, and moderate percentage of SOX17 expression pattern, thus suggesting that these cells are still in the early stage of DE. When the differentiated cells were cultured for 14 days, they were assessed for their capability of the formation of islet-like clusters by Diphenylthiocarbazone stain (DTZ). DTZ is a zinc-chelating substance that is known to selectively stain pancreatic β cells due to the high zinc content in these cells, of which is vital for packaging insulin in pancreatic β cells [27]. Our results revealed that BM-MSCs and SCAP are able to produce these islet-like clusters, as detected by DTZ. Moreover, to investigate if cells from both sources, differentiated by the two different methods, harbor an insulin-production ability, concentrations of secreted insulin and C-peptide were measured by ELISA after 14 days of induction. In our experiment, we found that the secretion of both markers was significantly enhanced by BM-MSCs and SCAP compared to the control groups, indicating a functional property of these cells.

Furthermore, the immunofluorescence staining of pancreatic cell markers—namely, but not limited to, C-peptide, glucagon, insulin and PDX-1 of both differentiated and undifferentiated cells—were also observed. Our results have demonstrated a high expression of these markers by all the differentiated BM-MSCs and SCAP, confirming their maturation. Our findings reflect what others have shown in their studies—PDX1 not only plays a crucial role in the early development of the pancreas, but it also takes part in sustaining the survival and mature phenotype in β cells [28,29]. Moreover, our data showed that the islet-like clusters originally isolated from bone marrow and apical papilla co-expressed insulin and glucagon, suggesting that the population of cells may be precursors to mature endocrine cell types, which was also reported by other investigators [30,31,32,33,34,35,36]. Additionally, the positive co-expression of insulin and C-peptide by the same cells confirms that proinsulin was synthesized by these cells and was not derived from any other resource [37].

## 4. Materials and Methods

### 4.1. Isolation of Mesenchymal Stem Cells (MSCs)

#### Subjects and Samples

Signed informed consent was obtained from all donors before inclusion in the study. Bone marrow aspirates and impacted immature third molar teeth were collected from six different donors who were healthy with no clinically evident disease, had not been taking any medication, non-smokers and non-alcohol consumers.

This study was approved by the institutional review board (IRB) at the Cell Therapy Center/University of Jordan (IRB-CTC/1-2020-03). All donors signed an informed consent form in accordance with the Helsinki declaration prior to sample collection.

### 4.2. Isolation and Expansion of Dental Stem Cells

The teeth were submerged in transport medium, consisting of an alpha modification of Eagle’s Medium (α-MEM; Gibco, Life Technologies, Carlsbad, CA, USA), supplemented with 10% penicillin/streptomycin (Invitrogen, Life Technologies, Carlsbad, CA, USA) and amphotericin B (Invitrogen, Life Technologies, Carlsbad, CA, USA), and stored at 4 °C until use.

Isolation of stem cells was performed within 24 h of receiving the sample. Stem cells from the apical papilla were isolated as previously described [38]. Briefly, the tissue was cut away from the immature roots by using a sterile scalpel and blade and then minced into small pieces. Small fragments were maintained in a culture medium of mesenchymal stem cells (MSCs); alpha MEM containing 10% Fetal Bovine Serum (FBS), 1% L-Glutamine and 1% Pencillin/streptomycin (Gibco, Carlsbad, CA, USA) were used and incubated at 37 °C in a 5% CO_2_ incubator. 

### 4.3. Isolation and Expansion of Bone Marrow Stem Cells

Human MSCs were isolated from BM aspirates as previously described [18]. Mononuclear cells (MNCs) were separated as previously described with some modifications [39] by the density gradient method; bone marrow aspirates were diluted with phosphate-buffered saline (PBS) pH 7.4 (Gibco, Carlsbad, CA, USA) in a 1:1 ratio, and then 10 mL of the diluted aspirate was gently transferred into 15 mL centrifuge tubes containing ficoll-paque (Sigma-Aldrich, Burlington, MA, USA). The tubes were then centrifuged at 350× *g* for 30 min with the break off. After centrifugation, the interfaces containing the MSCs were collected and placed in a 50 mL centrifuge tube. MNCs were washed by diluting the interface in a 1:5 ratio with PBS, Ph 7.4 and then centrifuged at 350× *g* for 8 min at 4 °C. After centrifugation, the supernatant was discarded and the pellet was suspended in a defined volume of complete cell culture medium alpha MEM containing 10% FBS, 1% L-Glutamine and 1% Pencillin/streptomycin (Gibco, Carlsbad, CA, USA).

MNCs were seeded at a density of 0.16–0.18 × 10^6^ cells/cm^2^ and incubated at 37 °C, 5% CO_2_ and 95% relative humidity. Cells were allowed to attach for 24 h then the medium and nonadherent cells were removed to be replaced with a fresh complete cell culture medium. On a daily basis, the cells were observed under an inverted microscope (Carl Zeiss, Oberkochen, Germany) and the culture medium was replaced every 3 days until individual colonies reached 70–80% confluence.

### 4.4. Characterization of MSCs by Flow Cytometry

In order to confirm the MSC identity of the derived cells, SCAP and BMSCs, the cells were immunophenotypically characterized by using a human mesenchymal stem cell characterization kit (BD stemflow kit, BD Biosciences, NJ, USA) and analyzed by flow cytometry.

Briefly, 1 × 10^6^ cells at P3 were collected using 0.25% Trypsin/EDTA (Gibco, CA, USA), then washed with PBS (Gibco, CA, USA). Following that, cells were incubated for 30 min at room temperature with the following fluorescin-labeled antibodies: FITC CD90, PE CD44, PerCP-Cy™5.5 CD105, APC CD73, and negative cocktail (which contains PE CD45, PE CD34, PE CD11b, PE CD19 and PE HLA-DR) and their isotype controls as per the manufacturer’s recommendations. After incubation, the cells were centrifuged at 300× *g* for 5 min, followed by re-suspension with PBS. The expression profile was analyzed by FACS DIVA software version 7 by using FACS Canto II (BD, Biosciences, NJ, USA).

### 4.5. Multilineage Differentiation of MSCs

To assess the differentiation potential of the derived SCAP and BMSCs, cells were induced to confirm their osteogenic and adipogenic differentiation potentials.

First, 1 × 10^5^ of MSCs at P3 were seeded on a 6-well plate (TPP, Zollstrasse, Trasadingen, Switzerland) in their complete growth media until reaching 60–70% confluence. After that, the growth medium was replaced by the differentiation medium, as cells were induced for their osteogenic and adipogenic differentiation using StemPro osteogenesis and adipogenesis differentiation kits (Gibco, Carlsbad, CA, USA), respectively. The medium was changed twice a week for a total of 3 weeks.

Uninduced cells maintained in their complete growth media for 3 weeks were used as the negative control.

### 4.6. Assessment of Differentiation

#### 4.6.1. Osteogenic Differentiation-Alizarin Red Stain

To confirm the osteogenic differentiation, Alizarin Red-S (ARS) staining was utilized to detect the presence of calcium deposition.

After removing the differentiation media and washing in PBS, the cells were fixed in 4% formaldehyde for 30 min and incubated for 20 min in 2% ARS pH 4.2 (Sigma Aldrich, Burlington, MA, USA). Stained calcium deposits were assessed using an Axio inverted light microscope (Ziess, Oberkochen, Germany). 

#### 4.6.2. Adipogenic Differentiation and Oil Red O Stain

For adipogenic differentiation, cells were stained to detect the formation of oil droplets.

After 3 weeks of differentiation, cells were washed with PBS and fixed with 4% formaldehyde for 30 min then incubated in a solution containing 0.3% of Oil Red Stain (Sigma Aldrich, Burlington, MA, USA) for 20 min. Stained oil droplets were observed under an axio inverted light microscope (Ziess, Oberkochen, Germany).

#### 4.6.3. Endoderm Differentiation (β Cells)

To produce the definitive endoderm layer as a starting point of beta cell differentiation, both SCAP and BMSCs cells were plated in 6-well plates until reaching 50–60% confluence, then the cell culture media was replaced by PSC Definitive Endoderm (DE) Induction media (Thermofisher, Waltham, MA, USA). At day 1, PSC Definitive Endoderm (DE) Induction medium A was added to the cells. At day 2, PSC Definitive Endoderm (DE) Induction media A was replaced with PSC Definitive Endoderm (DE) Induction medium B. At day 3, for the differentiation of the cells into a specific line of endoderm (Pancreatic differentiation), cells were induced by adding Advanced DMEM-F12 (Thermofisher, Waltham, MA, USA), supplemented with 1% BSA (Bovine serum albumin, Biowest, Nuaillé, France), 1% ITS (Insulin Transferrin selenium, Sigma, Burlington, MA, USA), 0.3 mM Taurine (Sigma, Burlington, MA, USA) and without serum for 2 days. At day 5, differentiating functional pancreatic cells were induced by Advanced DMEM-F12 (Thermofisher, Waltham, MA, USA), supplemented with 1.5% BSA (Biowest, Nuaillé, France), 1.5% ITS (Sigma, Burlington, MA, USA), 3 mM Taurine (Sigma, Burlington, MA, USA), 100 nM GLP (glucagon-like peptide Sigma, Burlington, MA, USA) and 1 mM nicotinamide (Sigma, Burlington, MA, USA) without serum for 10 days. The medium was exchanged every 2 days.

### 4.7. Validation of Endodermal Differentiation

#### Definitive Endoderm Markers: FoxA2 and SOX17

To determine the effectiveness of the differentiation into the definitive endoderm, FOXA2 and SOX17, which are the major markers of the definitive endoderm layer, were measured using flow cytometry.

At day 3 of definitive endoderm induction, both cell types were collected and fixed with cell fix 1× (BD Biosciences, Franklin Lakes, NJ, USA) for 15 min at room temperature, and were centrifuged for 5 min at 300× *g*. After that, cells were permeabilized by adding 500 μL of chilled absolute methanol for 10 min with vigorous vortexing and were incubated for 20 min with the following markers: SOX17-PE (BD, Biosciences, Franklin Lakes, NJ, USA) and FOXA2-Alexaflour647 (BD, Biosciences, Franklin Lakes, NJ, USA) at room temperature on the shaker. Antibodies were used at concentrations based on the manufacturer’s suggestion (BD, Biosciences, Franklin Lakes, NJ, USA). Following the incubation, 2 mL of cell wash (BD, Biosciences, Franklin Lakes, NJ, USA) was added to each tube with vigorous vortexing followed by centrifugation at 400× *g* for 5 min and resuspension with PBS. The expression profile was analyzed by Fluorescent activated cell sorter FACS Canto II (BD, Biosciences, Franklin Lakes, NJ, USA). Uninduced cells were used as a negative control.

### 4.8. Validation of the Formation and Maturation of Islet-like Clusters

#### Diphenylthiocarbazone Staining (DTZ)

To determine the maturity of the differentiated cells into islet-like clusters, on day 14 of differentiation, cells were fixed with 4% paraformaldehyde for 20 min, followed by a washing step with PBS. Then, cells were stained with Diphenylthiocarbazone (DTZ, Sigma, Massachusetts, USA) for 20 min. Finally, cells were observed under the inverted microscope to detect the production of stained islet-like clusters.

### 4.9. Immunofluorescence Staining

#### 4.9.1. Detection of Maturation Markers: Insulin, C-Peptide, Glucagon and PDX-1

At day 14 of differentiation, differentiated cells were examined to determine the maturity state of beta cell differentiation.

Induced and uninduced cells were plated onto coated coverslips, then fixed with 4% paraformaldehyde for 20 min. Afterwards, fixed cells were permeabilized with 0.25% Triton X for 15 min. Then, the blocking solution (Block Aid, Thermo scientific, Waltham, MA, USA) was added to the permeabilized cells for 1 h. Following that, cells were washed three times with washing buffer. The primary antibodies Anti-PDX-1 (host: rabbit) (Abcam), Anti-insulin (host: mouse), Anti-C peptide (host: mouse) and Anti-glucagon (host: rabbit) were added to different coverslips and incubated overnight at 4 °C. The next day, cells were washed three times with washing buffer. Then, the appropriate concentrations of secondary antibodies (AlexaFlour595, Anti-rabbit, Invitrogen), (AlexaFlour 488-Anti-rabbit, Invitrogen), (AlexaFlour 488-Anti-mouse, Invitrogen) and (AlexaFlour Anti-mouse, Invitrogen) were added and incubated for 1 h at 37 °C in a CO_2_ incubator. Then, the coverslips were stained with DAPI (Invitrogen/molecular probes) media for 5 min, then washed thrice with the washing buffer. Finally, coverslips were transferred onto glass slides loaded with one drop of Mounting media (DAKO, Santa Clara, CA, USA).

Uninduced cells were used as a negative control. Finally, the prepared slides were observed under a confocal microscope (Ziess, Oberkochen, Germany).

#### 4.9.2. Detection of Insulin and C-Peptide by ELISA

To evaluate the efficiency of beta cell differentiation for both cell types, the following markers, insulin and C-peptide (Abcam, Waltham, MA, USA), were measured at different time points of differentiation by collecting media at days 1, 7 and 14 of differentiation. The media were collected and stored at −80 °C until use. The measurements were carried out according to the manufacturer’s instructions.

### 4.10. Statistical Analysis

The data were analyzed using graphPad Prism and Microsoft Excel. All assays were performed in three independent experiments (n = 3), with triplicates of each. The results are expressed as mean ± standard deviation. Statistical significance was determined using the paired *t*-test and one-way ANOVA analysis. Significance was assigned for *p* < 0.05.

## 5. Conclusions

In the present study, the β-differentiation potential of BM-MSCs and SCAP was evaluated; our results revealed that SCAP had a higher differentiation ability into β-like cells compared to BM-MSCs. These findings demonstrated that cells isolated from either source are suitable candidates for the treatment of diabetes, regardless of their differentiation method. For future perspectives, in vivo studies are needed to establish the functionality and clinical potential of using generated IPCs within the diabetic mouse model.

## Data Availability

The datasets used and/or analyzed during the current study are available from the corresponding author.

## Abbreviations

ARS	Alizarin Red Stain
BM-MSCs	Bone-marrow-derived stem cells
BSA	Bovine serum albumin
DE	Definitive Endoderm
DM	Diabetes Mellitus
DTZ	Diphenythiocarbazone.
FBS	Fetal Bovine Serum
GLP-1	glucagon-like peptide-1
ICA	Islet-like cell aggregates (ICAs)
IPC	insulin-producing beta cells
IRB	Institutional review board
ISCT	International Society for Cellular Therapy
ITS	Insulin Transferrin selenium
MNC	Mononuclear cells
MSC	Mesenchymal stem cells
PBS	Phosphate buffered saline
SCAP	Stem cells of apical papilla
T1DM	Type 1 Diabetes Mellitus

## References

[1] Ackermann A.M., Gannon M. (2007). Molecular regulation of pancreatic β-cell mass development, maintenance, and expansion. J. Mol. Endocrinol..

[2] Chen C., Cohrs C.M., Stertmann J., Bozsak R., Speier S. (2017). Human beta cell mass and function in diabetes: Recent advances in knowledge and technologies to understand disease pathogenesis. Mol. Metab..

[3] Association A.D. (2010). Diagnosis and classification of diabetes mellitus. Diabetes Care.

[4] Shapiro A.J., Lakey J.R., Ryan E.A., Korbutt G.S., Toth E., Warnock G.L., Kneteman N.M., Rajotte R.V. (2000). Islet transplantation in seven patients with type 1 diabetes mellitus using a glucocorticoid-free immunosuppressive regimen. N. Engl. J. Med..

[5] Hering B.J., Clarke W.R., Bridges N.D., Eggerman T.L., Alejandro R., Bellin M.D., Chaloner K., Czarniecki C.W., Goldstein J.S., Hunsicker L.G. (2016). Phase 3 trial of transplantation of human islets in type 1 diabetes complicated by severe hypoglycemia. Diabetes Care.

[6] Zang L., Hao H., Liu J., Li Y., Han W., Mu Y. (2017). Mesenchymal stem cell therapy in type 2 diabetes mellitus. Diabetol. Metab. Syndr..

[7] Wan X.-X., Zhang D.-Y., Khan M.A., Zheng S.-Y., Hu X.-M., Zhang Q., Yang R.-H., Xiong K. (2022). Stem Cell Transplantation in the Treatment of Type 1 Diabetes Mellitus: From Insulin Replacement to Beta-Cell Replacement. Front. Endocrinol..

[8] Yang L., Li S., Hatch H., Ahrens K., Cornelius J.G., Petersen B.E., Peck A.B. (2002). In vitro trans-differentiation of adult hepatic stem cells into pancreatic endocrine hormone-producing cells. Proc. Natl. Acad. Sci. USA.

[9] Koblas T., Zacharovová K., Berková Z., Leontovic I., Dovolilová E., Zámecník L., Saudek F. (2009). In vivo differentiation of human umbilical cord blood-derived cells into insulin-producing beta cells. Folia Biol..

[10] Chen L.-B., Jiang X.-B., Yang L. (2004). Differentiation of rat marrow mesenchymal stem cells into pancreatic islet beta-cells. World J. Gastroenterol. WJG.

[11] Li J., Zhu L., Qu X., Li J., Lin R., Liao L., Wang J., Wang S., Xu Q., Zhao R.C. (2013). Stepwise differentiation of human adipose-derived mesenchymal stem cells toward definitive endoderm and pancreatic progenitor cells by mimicking pancreatic development in vivo. Stem Cells Dev..

[12] Xu B., Fan D., Zhao Y., Li J., Wang Z., Wang J., Wang X., Guan Z., Niu B. (2020). Three-dimensional culture promotes the differentiation of human dental pulp mesenchymal stem cells into insulin-producing cells for improving the diabetes therapy. Front. Pharmacol..

[13] Siqueira J., Lopes H. (1999). Mechanisms of antimicrobial activity of calcium hydroxide: A critical review. Int. Endod. J..

[14] Sun Z., Gou W., Kim D.-S., Dong X., Strange C., Tan Y., Adams D.B., Wang H. (2017). Adipose stem cell therapy mitigates chronic pancreatitis via differentiation into acinar-like cells in mice. Mol. Ther..

[15] Kawakubo K., Ohnishi S., Kuwatani M., Sakamoto N. (2018). Mesenchymal stem cell therapy for acute and chronic pancreatitis. J. Gastroenterol..

[16] Pavathuparambil Abdul Manaph N., Sivanathan K.N., Nitschke J., Zhou X.-F., Coates P.T., Drogemuller C.J. (2019). An overview on small molecule-induced differentiation of mesenchymal stem cells into beta cells for diabetic therapy. Stem Cell Res. Ther..

[17] Aydin S., Sağraç D., Şahin F. (2019). Differentiation Potential of Mesenchymal Stem Cells into Pancreatic β-Cells. Cell Biol. Transl. Med..

[18] Dominici M., Le Blanc K., Mueller I., Slaper-Cortenbach I., Marini F., Krause D., Deans R., Keating A., Prockop D., Horwitz E. (2006). Minimal criteria for defining multipotent mesenchymal stromal cells. The International Society for Cellular Therapy position statement. Cytotherapy.

[19] Zakrzewski W., Dobrzyński M., Szymonowicz M., Rybak Z. (2019). Stem cells: Past, present, and future. Stem Cell Res. Ther..

[20] Ullah I., Subbarao R.B., Rho G.J. (2015). Human mesenchymal stem cells-current trends and future prospective. Biosci. Rep..

[21] Elahi K.C., Klein G., Avci-Adali M., Sievert K.D., MacNeil S., Aicher W.K. (2016). Human mesenchymal stromal cells from different sources diverge in their expression of cell surface proteins and display distinct differentiation patterns. Stem Cells Int..

[22] Pittenger M.F., Discher D.E., Péault B.M., Phinney D.G., Hare J.M., Caplan A.I. (2019). Mesenchymal stem cell perspective: Cell biology to clinical progress. NPJ Regen. Med..

[23] Gabr M.M., Zakaria M.M., Refaie A.F., Abdel-Rahman E.A., Reda A.M., Ali S.S., Khater S.M., Ashamallah S.A., Ismail A.M., Ismail H.E.-D.A. (2017). From human mesenchymal stem cells to insulin-producing cells: Comparison between bone marrow-and adipose tissue-derived cells. BioMed Res. Int..

[24] Murry C.E., Keller G. (2008). Differentiation of embryonic stem cells to clinically relevant populations: Lessons from embryonic development. Cell.

[25] Halpern K.B., Vana T., Walker M.D. (2014). Paradoxical role of DNA methylation in activation of FoxA2 gene expression during endoderm development. J. Biol. Chem..

[26] Shimoda M., Kanai-Azuma M., Hara K., Miyazaki S., Kanai Y., Monden M., Miyazaki J.-I. (2007). Sox17 plays a substantial role in late-stage differentiation of the extraembryonic endoderm in vitro. J. Cell Sci..

[27] Shiroi A., Yoshikawa M., Yokota H., Fukui H., Ishizaka S., Tatsumi K., Takahashi Y. (2002). Identification of insulin-producing cells derived from embryonic stem cells by zinc-chelating dithizone. Stem Cells.

[28] Holland A.M., Hale M.A., Kagami H., Hammer R.E., MacDonald R.J. (2002). Experimental control of pancreatic development and maintenance. Proc. Natl. Acad. Sci. USA.

[29] Johnson J.D., Ahmed N.T., Luciani D.S., Han Z., Tran H., Fujita J., Misler S., Edlund H., Polonsky K.S. (2003). Increased islet apoptosis in Pdx1+/–mice. J. Clin. Investig..

[30] Alpert S., Hanahan D., Teitelman G. (1988). Hybrid insulin genes reveal a developmental lineage for pancreatic endocrine cells and imply a relationship with neurons. Cell.

[31] De Krijger R., Aanstoot H., Kranenburg G., Reinhard M., Visser W., Bruining G. (1992). The midgestational human fetal pancreas contains cells coexpressing islet hormones. Dev. Biol..

[32] Herrera P.-L., Huarte J., Zufferey R., Nichols A., Mermillod B., Philippe J., Muniesa P., Sanvito F., Orci L., Vassalli J.-D. (1994). Ablation of islet endocrine cells by targeted expression of hormone-promoter-driven toxigenes. Proc. Natl. Acad. Sci. USA.

[33] D’Amour K.A., Bang A.G., Eliazer S., Kelly O.G., Agulnick A.D., Smart N.G., Moorman M.A., Kroon E., Carpenter M.K., Baetge E.E. (2006). Production of pancreatic hormone–expressing endocrine cells from human embryonic stem cells. Nat. Biotechnol..

[34] Jiang J., Au M., Lu K., Eshpeter A., Korbutt G., Fisk G., Majumdar A.S. (2007). Generation of insulin-producing islet-like clusters from human embryonic stem cells. Stem Cells.

[35] Rezania A., Riedel M.J., Wideman R.D., Karanu F., Ao Z., Warnock G.L., Kieffer T.J. (2011). Production of functional glucagon-secreting α-cells from human embryonic stem cells. Diabetes.

[36] Riedel M., Asadi A., Wang R., Ao Z., Warnock G., Kieffer T. (2012). Immunohistochemical characterisation of cells co-producing insulin and glucagon in the developing human pancreas. Diabetologia.

[37] Gabr M.M., Zakaria M.M., Refaie A.F., Ismail A.M., Abou-El-Mahasen M.A., Ashamallah S.A., Khater S.M., El-Halawani S.M., Ibrahim R.Y., Uin G.S. (2013). Insulin-producing cells from adult human bone marrow mesenchymal stem cells control streptozotocin-induced diabetes in nude mice. Cell Transplant..

[38] Abuarqoub D., Awidi A., Abuharfeil N. (2015). Comparison of osteo/odontogenic differentiation of human adult dental pulp stem cells and stem cells from apical papilla in the presence of platelet lysate. Arch. Oral Biol..

[39] Gnecchi M., Melo L.G. (2009). Bone marrow-derived mesenchymal stem cells: Isolation, expansion, characterization, viral transduction, and production of conditioned medium. Stem Cells in Regenerative Medicine.

